# Effects of exogenous copper on microbial metabolic function and carbon use efficiency of *Panax notoginseng* planting soil

**DOI:** 10.3389/fmicb.2024.1390921

**Published:** 2024-07-10

**Authors:** Tong Wang, Xu Wang, Tarik Hadibi, Xun Ma, Haoyi Yao, Zhenya Tang, Fangling Fan, Yizong Huang

**Affiliations:** ^1^School of Energy and Environment Science, Yunnan Normal University, Kunming, China; ^2^Key Laboratory of Solar Heating and Cooling Technology of Yunnan Provincial Universities, Kunming, China; ^3^Faculty of Modern Agricultural Engineering, Kunming University of Science and Technology, Kunming, China

**Keywords:** exogenous copper, *Panax notoginseng*, carbon source utilization, extracellular enzyme activities, carbon use efficiency

## Abstract

Soil copper (Cu) pollution is a serious environmental risk in the *Panax notoginseng* planting area. However, the effect of Cu on soil microbial metabolism and nutrient cycling in this area remains unknown. Therefore, Biolog ECO-plate and enzyme stoichiometry methods were utilized in this study to investigate the impact of exogenous Cu (control: 0 mg·kg^−1^; Cu100: 100 mg·kg^−1^; Cu400: 400 mg·kg^−1^; and Cu600: 600 mg·kg^−1^) on the metabolic function of soil microbial and nutrient limitation in the *P. notoginseng* soil. The results indicated that Cu100 significantly increased soil organic carbon (SOC), total phosphorus (TP), soil C:N, microbial biomass carbon (MBC), and microbial biomass nitrogen (MBN) 9.89%, 15.65%, 17.91%, 61.87%, and 90.56% higher than the control, respectively. Moreover, the carbon source utilization ratio of carbohydrates, amino acids, and amphiphilic compounds of Cu100 also increased by 7.16%, 25.47%, and 84.68%, respectively, compared with the control. The activities of β-1,4-glucosidase, cellobiohyrolase, leucine amino peptidase, β-1,4-N-acetylglucosaminidase, and phosphatase significantly decreased with increasing Cu concentration. Soil enzyme stoichiometry showed that all treatments were limited by nitrogen (vector angle < 45°; 19.045–22.081). Cu600 led to the lowest carbon limitation (1.798) and highest carbon use efficiency (CUE:0.267). The PLS-SEM model also showed that MBC, MBN, MBP, and microbial diversity positively affected carbon and nitrogen limitation (0.654 and 0.424). Soil carbon, nitrogen, phosphorus, stoichiometric ratio, MBC, MBN, and MBP positively affected CUE (0.527 and 0.589). The microbial diversity index significantly negatively affected CUE (−1.490). Multiple linear stepwise regression analyses showed that CUE was mainly influenced by MBC, AP, C:P, and LAP. Thus, *P. notoginseng* soil can benefit soil microbial carbon and nitrogen limitations at low Cu concentrations. Clarifying the metabolic activity and nutritional status of microorganisms under Cu stress can provide some theoretical basis for realizing China's comprehensive and effective management and control policies for environmental risks from metals by 2035.

## 1 Introduction

Soil heavy metal pollution is one of the most serious environmental issues, exposing severe implications for human health, ecosystem function, and food security (Peng et al., 2022). In China, heavy metal contamination in basic farmland conservation area has been reported to exceed standards by as much as 16.1% (Wen et al., 2022). Wenshan, China, serves as a primary production area for *Panax notoginseng* and is also renowned for its mining activities (Li et al., 2014; Ou et al., 2016). Unfortunately, the soil in this region contains various heavy metals, including copper (Cu), cadmium (Cd), chromium (Cr), arsenic (As), and mercury (Hg), which often surpass China's soil environmental quality standards (Huang et al., 2023). The unregulated expansion of mining areas and the widespread use of heavy-metal pesticides have exacerbated heavy-metal pollution (Duan et al., 2015; Wu et al., 2022). Consequently, this situation poses critical threats to soil quality in such regions and the safety of *P. notoginseng* products bound for human consumption (Duan et al., 2015). Of those heavy metals, Cu pollution is in the soil of the highly coveted and irreplaceable planting regions (Zeng et al., 2009). Recent studies reveal that soil Cu content in the soil of *P. notoginseng* planting areas in Wenshan County ranges from 29.16 to 285.48 mg·kg^−1^ (Zu et al., 2017), which exceeds 49.9% of the standard limits set in the “Soil Environmental Quality Risk control standard for soil contamination of agricultural land (GB 15618, 2018)” of China, which specifies a limit of 100 mg·kg^−1^ for soil Cu (GB 15618, 2018).

Cu is an essential trace element for crop growth and microbial development (Gilbert, 1952). At optimal concentrations, it stimulates soil microbial growth and enhances the abundance of soil microbial communities (Mir et al., 2021). However, exceeding a certain Cu concentration threshold can negatively impact photosynthesis, microbial activities, enzyme function, and antioxidant systems, ultimately affecting crop growth and altering the abundance, structure, and function of microbial communities (Gong et al., 2019; Karimi et al., 2021).

Microorganisms play a crucial role in nutrient cycling and the transformation of organic matter within soil systems (Coonan et al., 2020; Philippot et al., 2023). Under heavy metal stress, soil microorganisms divert the energy used for community expansion to maintain cell function (Duan et al., 2022). In addition, the toxicity of heavy metals reduces the energy utilization efficiency of microbial metabolism, and more carbon sources are required to maintain microbial activity, thereby reducing microbial biomass (Graham and Haynes, 2005). Notably, soil Cu pollution inhibits enzyme activity, disrupting the cyclic transformation of major nutrients (Asensio et al., 2013). The roots of *Panax notoginseng* secrete various root exudates, including sugars, carboxylic acids, and amino acids, into the soil. These exudates can alter the balance of the *P. notoginseng* soil microbial community (Zhang et al., 2019; Zhao et al., 2023). Long-term cultivation of *P. notoginseng* has been observed to shift the dominant microbial community from bacteria to fungi (Zhang et al., 2019). Additionally, soil microorganisms are also affected by heavy metals in the soil. Research on heavy metal pollution in *P. notoginseng* planting soil primarily focuses on the present polluted status investigation, plant absorption and accumulation, and human health risk assessments. The specific impacts of Cu stress on nutrient cycling and carbon metabolism on *P. notoginseng* soil remain largely unexplored and warrant further investigation.

Recent studies have highlighted the importance of extracellular enzyme stoichiometry (C:N:P) as an effective measure for evaluating soil microbial energy and nutrient limitations (Kumar and Ghoshal, 2017; McGee et al., 2019). Furthermore, carbon use efficiency (CUE) provides valuable insights into variations in microbial abundance, the rate of soil carbon storage turnover, and the intensity and activity of microbial metabolism (Sinsabaugh et al., 2013; Mooshammer et al., 2017; Domeignoz-Horta et al., 2020). To address these gaps, our study utilizes BIOLOG technology to examine the carbon source utilization metabolic characteristics of soil microorganisms in *P. notoginseng* plantation soil under exogenous Cu stress. We also explore the effects of Cu on carbon, nitrogen, and phosphorus limitations in soil microbes. Ultimately, this research aims to establish a theoretical foundation for ecological risk assessment and microbial early warning systems related to Cu pollution in *P. notoginseng* plantation soil.

## 2 Materials and methods

### 2.1 Experimental site and soil sampling

The experimental soil was collected from the Miaoxiang *Panax notoginseng* Scientific Experimental Site in Wenshan Prefecture, Yunnan Province, China (23°31′44″N, 104°19′13′″E). The region experiences a subtropical monsoon climate with an average annual temperature of 16.6°C and annual cumulative rainfall of 1,111 mm. The soil in this study was yellowish-brown lateritic soil developed from tertiary igneous rocks. On June 5, 2022, soil samples were cored from plots where *P. notoginseng* had been planted for over 2 years. Three random plots, spaced 10 meters apart (each measuring 0.5 m × 0.5 m), were selected. The top 1 cm of surface soil was removed, and soil from 1 to 20 cm depth was cored. The collected soil samples were homogenized and sieved through a 2 mm mesh to remove impurities. A fraction of the sample was kept under cold storage at 4°C for further analysis of extracellular enzyme activity, microbial carbon source metabolism, microbial biomass carbon, nitrogen, and phosphorus. Another portion of the samples was used to evaluate the fundamental physicochemical properties after air drying. Soil bulk density samples were also collected, and each sampling site was repeated three times. The total Cu content of the background soil was 26.07 mg·kg^−1^.

### 2.2 Experimental design

An incubation experiment including three exogenous Cu treatments (Cu100, Cu400, and Cu600) and a control was designed. Exogenous Cu was not added to the control. The CuSO_4_ solution was added to the soil to obtain total Cu concentrations of 100 mg·kg^−1^ for the Cu100 treatment, 400 mg·kg^−1^ for the Cu400 treatment, and 600 mg·kg^−1^ for the Cu600 treatment. The homogenized soil samples were placed in plastic pots and sealed using a fibrous membrane to ensure adequate ventilation while minimizing moisture evaporation. Incubation conditions were maintained at a constant temperature of 25°C and a consistent water content of 25%. The fibrous membrane on the top of the pots was removed after 7 days, and 1-year-old *P. notoginseng* seedlings were transplanted into the pots. Three seedlings were planted in each pot. Following a 30-day incubation period, the soil microbial properties were analyzed, and seedling survival rates were investigated.

### 2.3 Soil testing methods

Soil bulk density was determined using the ring knife method. Soil pH was measured using an electrode method with a soil-water ratio of 1:2.5 (Rayment and Higginson, 1992). The soil microbial biomass carbon and nitrogen content was also determined using the chloroform fumigation-potassium sulfate extraction method. The microbial biomass phosphorus was estimated using the chloroform fumigation-sodium bicarbonate extraction method. The SOC was quantified using the K_2_Cr_2_O_7_-FeSO_4_ titration method (Vance et al., 1987), while the total nitrogen was analyzed by H_2_SO_4_-HClO_4_ digestion followed by automated intermittent element analysis (AMS-Alliance) (Khan et al., 2001). The total phosphorus in the soil was measured utilizing the H_2_SO_4_-HClO_4_ digestion-molybdenum antimony anti-colorimetric method (Olsen, 1954). Furthermore, ammonium nitrogen and nitrate nitrogen were determined through the colorimetric method using a flow analyzer (SEAL Analytical) (Khan et al., 2001), while available phosphorus was estimated via the molybdenum antimony anti-colorimetric method (Olsen, 1954). The seedling survival rate of *Panax notoginseng* was at 75% of control, 87.5% of T1, 62.5% of T2, and 62.5% of T3. The results of the basic physical and chemical properties of the soil are shown in Table 1.

**Table 1:** The basic physical and chemical properties of the soil.

**pH**	**EC**	**${\text{NH}}_{4}^{+}$-N**	**${\text{NO}}_{3}^{-}$-N**	**AP**	**Cu background value**	**SOC**	**TN**	**Bulk density**
	**ms**·**cm**^−1^	**mg**·**kg**^−1^	**mg**·**kg**^−1^	**mg**·**kg**^−1^	**mg**·**kg**^−1^	**g**·**kg**^−1^	**g**·**kg**^−1^	**g**·**cm**^−3^
7.18	0.10	7.37	11.17	44.95	26.07	20.17	1.82	1.32

The BIOLOG-ECO plate method was employed to evaluate soil microbial carbon metabolism with 31 distinct carbon sources, each with three replicated samples. Samples of 10 g of soil were suspended in 90 ml of an aseptic of 0.85% NaCl solution and subjected to agitation at 180 rpm in a constant-temperature shaker for 30 min. After static incubation for 15 min, the supernatants were collected and subjected to subsequent continuous dilution procedures. A diluted solution that had been adjusted to a 10^−3^ times concentration was used to inoculate the BIOLOG-ECO microplates, which were then placed at 28°C for an incubation period of 10 days. The absorbance level (OD) was measured at 590 nm using a spectrophotometer (SPECTROstar Nano) every 24 h.

Soil extracellular enzyme activities were determined using fluorometric measurements (Bell et al., 2013), and the activities of five extracellular enzymes were measured. The enzyme species were β-1, 4-glucosidase (BG), cellobiohydrolase (CBH), leucine amino peptidase (LAP), β-1, 4-N-acetylglucosaminidase (NAG), and phosphatase (AP). The substrates were 4-methylumbelliferyl-β-D-glucoside, 4-methylumbelliferyl-β-D-cellobioside, L-leucine-7-amido-4-methylcoumarin hydrochloride, 4-methylumbelliferyl-N-acetyl-β-D-glucosaminide, and 4-methylumbelliferyl-phosphate, respectively. The fluorescence spectrophotometer was used to determine the excitation wavelength at 365 nm and the emission wavelength at 450 nm.

### 2.4 Statistical analysis

The average well color development (AWCD), which represents the capacity of culturable microbes to consume various substrates (carbon sources), is used to depict the metabolic activity of the soil microbial population. The AWCD was calculated by Equation (1) follows (Garland, 1996):


(1) $$\begin{array}{l} AWCD=\sum \left(Ci-r\right)/31 \end{array}$$

where AWCD means average well color development, is the absorbance at 590 nm of each reaction well, *Ci* represents the absorbance values of the 31 carbon-containing wells on the BIOLOG-ECO plate, and *r* is the comparable absorbance of the control well (water in the control well); negative (C*i*-r) values were set to zero.

Soil microbial community richness index *S* is expressed by the number of reactive wells (absorbance value > 0.25, meaning the well's carbon source is absorbed and utilized, and the well is a reactive pore).

The Shannon-Wiener index (*H*) of the soil microbial community represents the functional diversity of the microbial community. The *H* parameter is given as follows Equation (2) (Garland, 1996):


(2) $$\begin{array}{l} H=-\sum \left(Pi\times \ln\;Pi\right) \end{array}$$

where *Pi* = (C*i* – r)/∑(C*i* – r). *Pi* represents the ratio of the relative absorbance value of the *i*-th well on the BIOLOG-ECO plate to the sum of the average relative absorbance values across all wells on the plate.

The soil microbial community evenness index Pielou (E) is expressed as the ratio of the Shannon-Wiener index of the microbial community to the logarithm of the richness index. The Pielou (E) is given using Equation (3):


(3) $$\begin{array}{l} E=H/\ln\;S \end{array}$$

where *S* is the richness index.

The Simpson index (D) of soil microbial communities, also known as the dominance index, is a measure of concentration in terms of diversity. The Simpson index (D) can be calculated using Equation (4) as follows:


(4) $$\begin{array}{l} D=1-\sum Pi^{2} \end{array}$$

The EEA is expressed in nmol·(g·h)^−1^. However, the enzyme stoichiometry ratios of EEAC/N, EEAC/P, and EEAN/P are calculated using [(BG + CBH)/(NAG + LAP)], [(BG + CBH)/AP], and [(NAG + LAP)/AP], respectively. The vector lengths (carbon limitation) and angles (vector angle < 45° for nitrogen limitation and >45° for phosphorus limitation) were calculated for all treatments (Deng et al., 2019). The vector length is the square root of the sum of the squares of *x* and *y* (Equation 5). Where x represents the relative activity of C (BG and CBH) and P (AP) to acquire enzymes, and y represents the relative activity of C and N (NAG and LAP) to acquire enzymes. The vector length and angle of the enzyme stoichiometry ratio were used to quantify the extent of microbial carbon, nitrogen, or phosphorus limitation. A longer vector length indicates a greater degree of carbon limitation, while vector angles < 45° and >45° represent nitrogen and phosphorus limitation, respectively, with a stronger extent of limitation indicated by a larger deviation (Deng et al., 2019).


(5) $$\begin{array}{l} Vector\;length:\;L=\sqrt[{2}]{x^{2}+y^{2}} \end{array}$$

Where the vector angle is the arctangent of the straight line from the origin to point (*x, y*) and can be defined using Equation (6).


(6) $$\begin{array}{l} Vector\;Angle\;\left(A\right)\;=\;Degrees\;\left[Atan2\left(x,y\right)\right] \end{array}$$

The carbon use efficiency (CUE) is given using Equation (7) (Sinsabaugh et al., 2013):


(7) $$\begin{array}{l} CUE=CUE_{\max}\times {\left\{\left(S_{C:N}\times S_{C:P}\right)÷\left[\left(K_{C:N}+S_{C:N}\right)\times \left(K_{C:P}+S_{C:P}\right)\right]\right\}}^{0.5} \end{array}$$

Where S_C:N_ = B_C:N_/L_C:N_ × 1/EEA_C:N_, S_C:P_ = B_C:P_/L_C:P_ × 1/EEA_C:P_, L_C:N_ is the SOC: TN, L_C:P_ is the SOC: TP, B_C:N_ is the MBC: MBN, and B_C:P_ is the MBC: MBP. K_C:N_ and K_C:P_ are the half-saturation constants of CUE based on the availability of C, N, and P. The model assumes that the maximum growth rate occurs when the proportion of absorbable nutrients supply matches the microbial biomass stoichiometry and the growth efficiency is proportional to the geometric mean ratio of N and P supply relative to C. It is assumed that all models' K_C:N_ and K_C:P_ are 0.5, and CUE_max_ is 0.6 (Sinsabaugh et al., 2013).

One-way analysis of variance (ANOVA) was used with Duncan's test to evaluate significant differences (*p* < 0.05) in soil among different Cu concentrations. The final data were expressed as means ± standard deviation. Multiple linear stepwise regression analysis and model establishment were performed by SPSS 18.0. The absorbance values at 168 h of culturing period were selected to analyze carbon source metabolism characteristics and soil microbial diversity indices. Graphs were introduced by OriginPro2021. RDA analysis and corresponding graphs were conducted using Canoco5. Smartpls4 was employed to select a PLS-SEM model to assess further potential pathways involved in carbon-nitrogen limitation and carbon utilization efficiency.

## 3 Results

### 3.1 Effect of exogenous copper on basic physicochemical properties of soil

Table 2 presents the basic physical and chemical properties of the soil. Except for total nitrogen, Cu addition significantly impacted microbial biomass carbon (MBC), microbial biomass nitrogen (MBN), and microbial biomass phosphorus (MBP). Specifically, in the Cu100 treatment, soil organic carbon (SOC), total phosphorus (TP), MBC, MBN, and the C:N ratio were significantly higher than those in the control and other treatments (*p* < 0.05). However, the soil C:P and N:P ratios in Cu100 (39.95 ± 1.99 and 3.25 ± 0.24, respectively) were the lowest among all treatments. The highest soil C:P and N:P ratios were observed in the Cu600 treatment, with soil C:P ratios at 47.50 and N:P ratios at 4.34.

**Table 2:** Soil physical and chemical properties under different copper application amounts.

**Treatment**		**Control**	**Cu100**	**Cu400**	**Cu600**
SOC	g·kg^−1^	18.91 ± 0.81b	20.78 ± 0.33a	19.12 ± 0.15b	19.34 ± 0.27b
TN	g·kg^−1^	1.81 ± 0.02a	1.69 ± 0.11a	1.74 ± 0.01a	1.77 ± 0.09a
TP	g·kg^−1^	0.45 ± 0.01b	0.52 ± 0.03a	0.43 ± 0.02b	0.41 ± 0.03b
C:N		10.44 ± 0.34b	12.31 ± 0.64a	11.01 ± 0.13b	10.95 ± 0.44b
C:P		42.39 ± 1.62ab	39.95 ± 1.99b	45.03 ± 2.29ab	47.50 ± 3.53a
N:P		4.06 ± 0.07a	3.25 ± 0.24b	4.09 ± 0.19a	4.34 ± 0.28a
pH		7.37 ± 0.04a	7.11 ± 0.07b	6.45 ± 0.04c	6.22 ± 0.08d
MBC	mg·kg^−1^	119.65 ± 8.69b	193.68 ± 10.74a	117.90 ± 7.74b	113.33 ± 5.59b
MBN	mg·kg^−1^	6.46 ± 0.88b	12.23 ± 1.36a	4.53 ± 0.32bc	3.99 ± 0.73c
MBP	mg·kg^−1^	16.45 ± 0.33a	14.63 ± 0.66b	11.93 ± 0.66c	10.07 ± 0.33d

Different letters in the same row indicate significant differences between treatments (p <0.05).

### 3.2 The effects of exogenous copper on soil microbial metabolic activity and carbon source utilization characteristics

**Figure 1:**
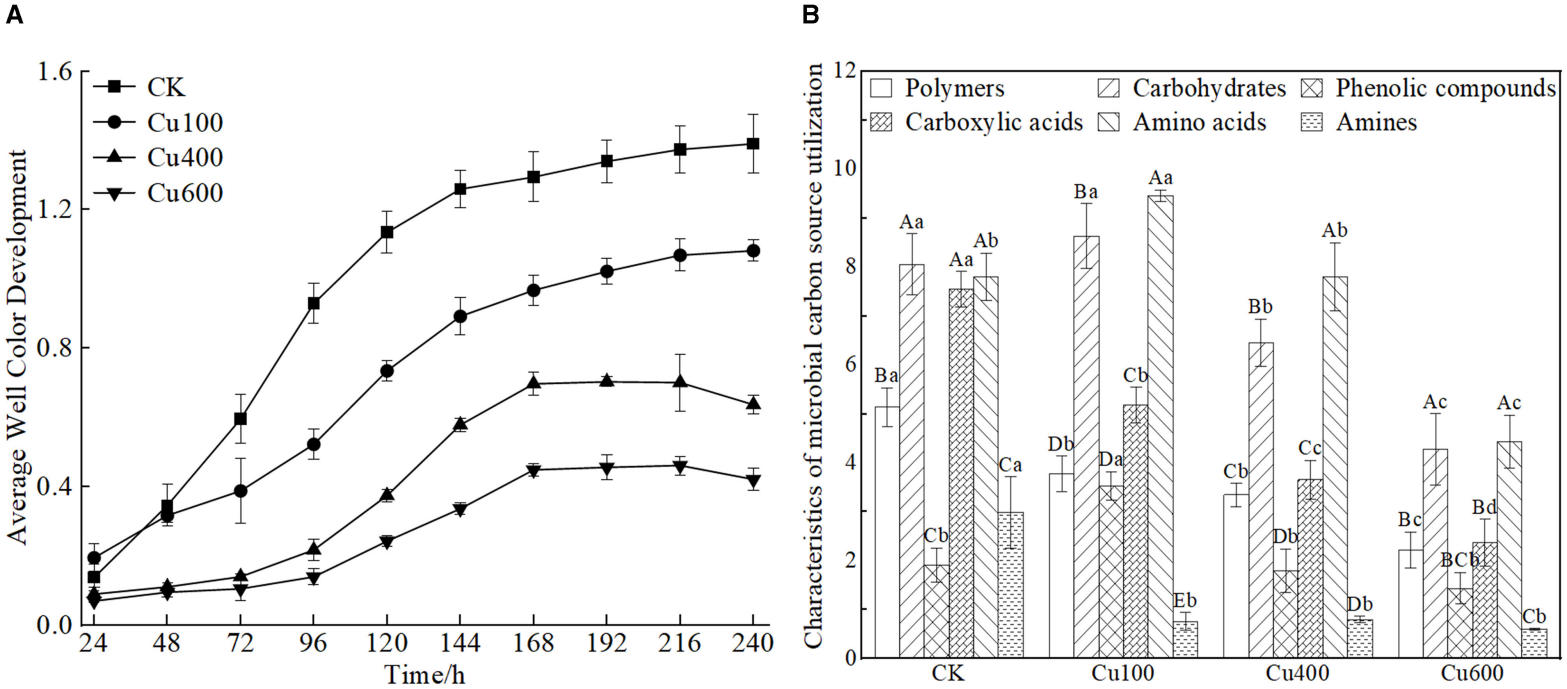
Changes of AWCD value of soil microbial carbon sources **(A)** and utilization characteristics of six types of carbon sources under Cu treatment **(B)**. In **(B)**, different uppercase letters indicate significant differences in different types of carbon sources in the same treatment group, and different lowercase letters indicate significant differences between groups of the same type of carbon sources (*p* < 0.05).

The average well color development (AWCD) values for the control and Cu100 treatments increased rapidly after 24 h of cultivation (Figure 1A). However, the increase in the Cu100 treatment was lower than that in the control. In the Cu400 and Cu600 treatments, AWCD values increased rapidly after 72 h, and microbial utilization of carbon sources across all treatments stabilized after 168 h. Variance analysis revealed that the AWCD value of the control was significantly higher than that of other treatments starting from 72 h (*p* < 0.05), with increases of 53.6%, 352.7%, and 467.6% compared to Cu100, Cu400, and Cu600, respectively.

The relative utilization rate of different carbon sources by the soil microbial community in Cu-treated soil differed significantly from that in the control (Figure 1B). Overall, soil microbes exhibited the following trend in their ability to utilize major carbon sources: amino acids > carbohydrates > carboxylic acids > polymers > hydrophobic compounds > amines. The Cu100 treatment exhibited the highest utilization rates of carbohydrates (8.631 ± 0.663) and amino acids (9.457 ± 0.116), with increases of 7.16 and 25.5% compared to the control, respectively. Conversely, the utilization rate of hydrophobic compounds in the Cu100 treatment (3.533 ± 0.288) was significantly higher than that in other treatments (*p* < 0.05). Carboxylic acid and polymer carbon source utilization by soil microbes decreased significantly with increasing soil Cu concentration (p <0.05).

### 3.3 Effects of exogenous copper on microbial community structure diversity

The richness index (S) of soil microbes in the control (41.90) was significantly higher than that in the other treatments (Table 3). As exogenous Cu concentration increased, the richness index decreased significantly from 30.68 in the Cu100 to 13.06 in the Cu600 treatment. However, the Shannon-Wiener index did not differ significantly among treatments, with an average value of 3.13. The Simpson index of the Cu600 treatment (0.94) was lower than that of the control (0.96) and Cu100 treatments (0.96) but did not show a significant difference with that of Cu400. Pielou's evenness index (E) was significantly higher in the Cu400 and Cu600 treatments compared to the Control and Cu100 treatments (*p* < 0.05).

**Table 3:** Soil microbial diversity index.

**Treatment**	**Richness index (*S*)**	**Shannon-Wiener index (*H*)**	**Simpson index (*D*)**	**Pielou index (*E*)**
Control	41.90 ± 1.85a	3.13 ± 0.04a	0.956 ± 0.01ab	0.838 ± 0.02c
Cu100	30.68 ± 1.80b	3.22 ± 0.05a	0.959 ± 0.01a	0.929 ± 0.02c
Cu400	21.62 ± 1.04c	3.11 ± 0.06a	0.944 ± 0.01abc	1.022 ± 0.02a
Cu600	13.06 ± 1.74d	3.08 ± 0.05a	0.939 ± 0.01c	1.205 ± 0.08b

Different letters in the same column indicate significant differences using ANOVA at p <0.05.

### 3.4 Effects of soil extracellular enzyme activity and enzymatic stoichiometry

**Figure 2:**
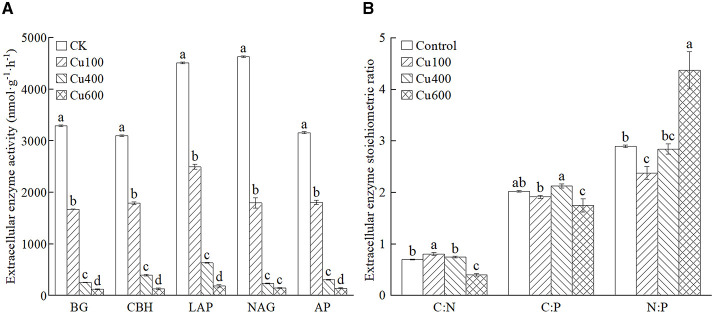
**(A, B)** Extracellular enzyme activity and stoichiometry ratio of different treatments. Different letters in the same column indicate significant differences between treatments using ANOVA at *p* < 0.05.

The extracellular enzyme activity (EEAs) in the soil treated with exogenous Cu was significantly lower than that in the control (*p* < 0.05; Figure 2A). This decrease became more pronounced with increasing Cu concentration. Among all treatments, the Cu600 treatment exhibited the lowest EEAs, accounting for 0.88%−2.40% of the EEAs in the control. Notably, the activities of carbon acquisition enzymes (BG and CBH) and nitrogen acquisition enzymes (NAG and LAP) were higher than those of phosphorus acquisition enzymes (AP) in all treatments (Figure 2A).

The C:N of extracellular enzymatic stoichiometry ratios in all treatments were in the range of 0.402–0.808, C:P was 1.753–2.127, and N:P was 2.377–4.374 (Figure 2B). Both C:P and N:P ratios exceeded 1 in all treatments. The addition of exogenous Cu led to variations in these ratios. Specifically, the Cu100 treatment promoted a significantly higher C:N ratio (0.878) compared to other treatments (*p* < 0.05). The Cu400 treatment exhibited the highest C:P ratio (2.127), significantly surpassing that of the Cu600 treatment (*p* < 0.05). Conversely, the Cu600 treatment resulted in a significantly higher N:P ratio (4.374) than other treatments.

### 3.5 Effects of ecological enzymatic stoichiometric vectors and carbon use efficiency

The vector length of the Cu400 treatment was significantly higher than that of the Cu100 and Cu600 treatment (*p* < 0.05). Specifically, Cu100 and Cu600 decreased by 2.94 and 16.10%, respectively, compared to the control; Cu400 increased by 5.23% compared to the control (Table 4). The enzyme stoichiometric vector analysis revealed that all treatments had vector angles <45°, indicating nitrogen limitation for soil microbes in *P. notoginseng* planting soil. Notably, the vector angle of the Cu100 treatment was significantly higher (15.9%) than that of the control treatment (*p* < 0.05). Conversely, the vector angle of the Cu600 treatment was significantly lower (32.0%) than that of the control treatment (*p* < 0.05). The carbon use efficiency value for treatments with exogenous Cu was higher by 0.021–0.058 than the control. Specifically, Cu100, Cu400, and Cu600 treatments exhibited CUE values 27.3%, 10.05%, and 27.8% higher than the control (*p* < 0.05), respectively.

**Table 4:** Vector variation characteristics of soil enzyme stoichiometry of different treatments.

**Treatment**	**Vector length**	**Vector angle**	**CUE**
Control	2.14 ± 0.02ab	19.05 ± 0.16b	0.21 ± 0.01b
Cu100	2.08 ± 0.02b	22.08 ± 1.11a	0.27 ± 0.02a
Cu400	2.26 ± 0.03a	19.38 ± 0.62b	0.23 ± 0.00b
Cu600	1.80 ± 0.13c	12.95 ± 0.98c	0.27 ± 0.02a

Different letters in the same column indicate significant differences between treatments (p <0.05).

### 3.6 RDA analysis of the effects of exogenous copper on soil microbial carbon and nitrogen limitation and carbon use efficiency

**Figure 3:**
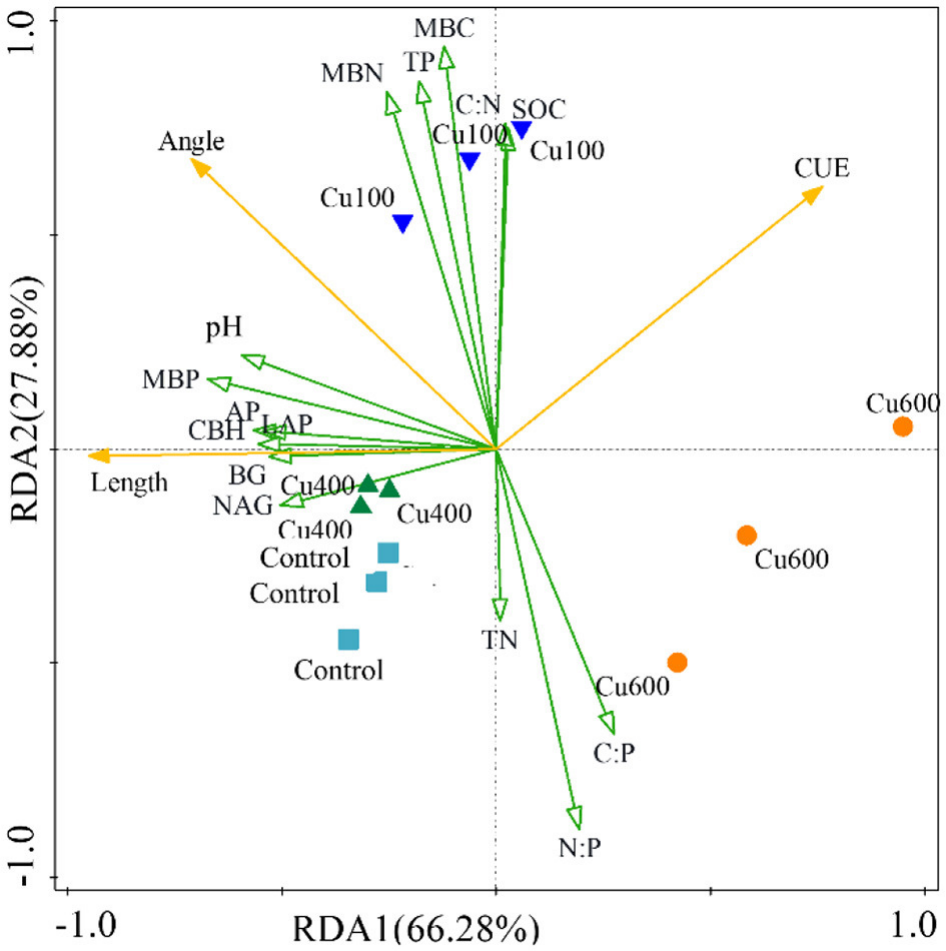
Redundant analysis of carbon and nitrogen limits and CUE by environmental factors under different treatments.

The RDA analysis was used to analyze the effects of exogenous Cu on soil microbial carbon and nitrogen limitation, as well as carbon use efficiency (Figure 3). The RDA1 and RDA2 accounted for 94.16% of the variation in extracellular enzyme activities (BG, CBH, LAP, NAG, and AP), CUE, and the other selected variations. The results showed the carbon limitation (vector length) was mainly and positively influenced by extracellular enzyme activity, pH, and MBP. The nitrogen limitation (vector angle) was mainly and positively influenced by extracellular enzyme activity, MBC, MBN, MBP, TP, C:N, pH, and SOC, but negatively affected by TN, C:P, and N:P. The CUE was negatively affected by pH, MBP, and EEA factors. Moreover, the CUE value was negatively correlated with carbon limitation and not with nitrogen limitation due to the right angle. In summary, the results of the RDA analysis indicated that environmental factors play a vital role in adjusting soil microbial functions and nutrient cycling processes in *P. notoginseng* planting soil. Specifically, EEAs, pH, MBP, TN, C:N, and N:P ratios contribute to differences in carbon and nitrogen limitation. Nevertheless, pH, MBP, and EEAs exhibit detrimental impacts on the CUE.

### 3.7 The effects of soil environmental factors on carbon and nitrogen limitations and CUE

**Figure 4:**
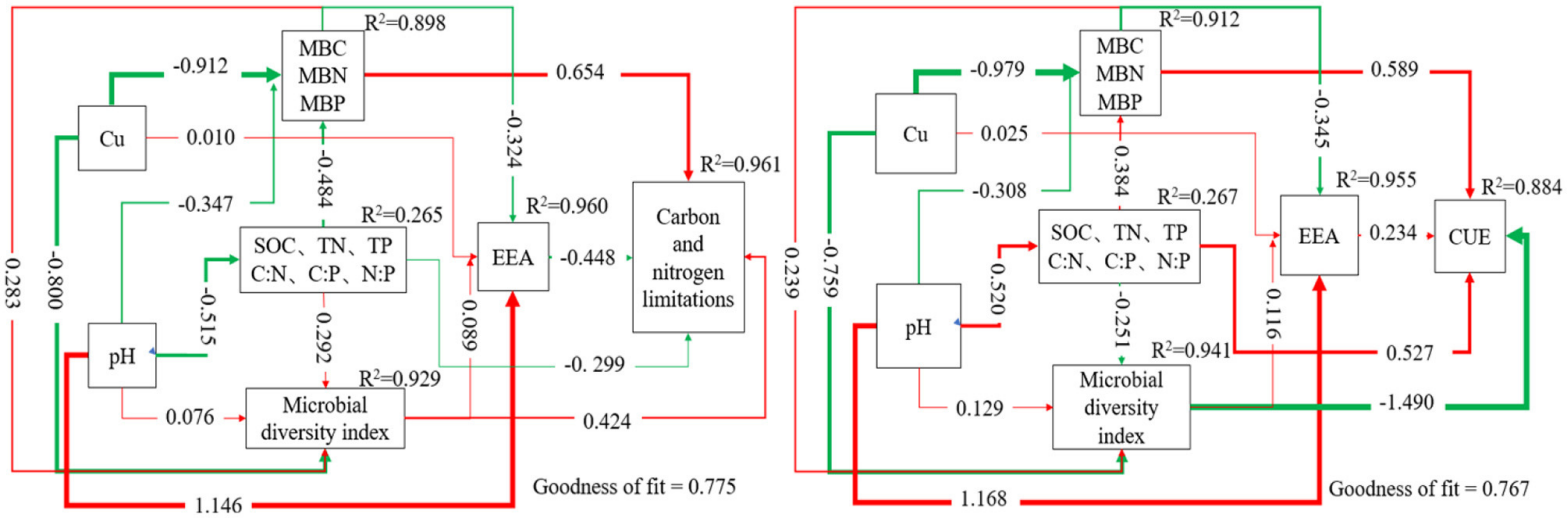
Partial least squares path modeling model. PLS-SEM path modeling shows the direct and indirect effects of Cu, soil physical and chemical properties, microbial biomass, microbial diversity, and extracellular enzymes on soil carbon and nitrogen limitation, CUE, and utilization of different types of carbon sources. The red and green lines represent positive and negative causal relationships. The numbers on the arrows indicate the standardized path coefficients; *R*^2^ represents the variance of the dependent variables explained by the model.

Partial Least Squares Structural Equation Modeling (PLS-SEM) was used to further analyze the effects of soil microbial biomass, environmental factor variables, EEAs, and microbial diversity indices on soil carbon and nitrogen limitations, as well as CUE (Figure 4). The analysis demonstrated that MBC, MBN, MBP, and microbial diversity exhibited substantial positive contributions to carbon and nitrogen constraints with path coefficients of 0.654 and 0.424, respectively. In contrast, extracellular enzyme activity negatively affected carbon and nitrogen constraints (−0.448), while soil SOC, TN, TP, and their stoichiometric ratios exerted a minor negative effect on carbon and nitrogen constraints (−0.299). MBC, MBN, MBP, and soil SOC, TN, TP, and their stoichiometric ratios presented positive effects on CUE with the path coefficients 0.589 and 0.527, respectively. However, microbial diversity had a negative impact on CUE with a path coefficient of −1.490, and extracellular enzyme activity contributed to a slightly positive impact on CUE with a path coefficient of 0.234. Multiple linear stepwise regression analysis (Table 5) showed that MBC, AP, C:P, and LAP could explain 98.2% of the variation of CUE, Pielou index could only explain 29.6% of the variation of carbon limitation, and MBN could explain 62.9% of the variation of nitrogen limitation.

**Table 5:** CUE, carbon, and nitrogen limitation's multiple linear stepwise regression model.

**Model**	***R* ^2^**	***F***	***p***
CUE = 0.941^*^MBC-5.084^*^AP-0.315^*^C:P + 4.460^*^LAP	0.982	149.863	<0.01
Length = −0.600^*^Pielou	0.296	5.616	<0.05
Degree = 0.814^*^MBN	0.629	19.627	<0.01

Length stands for carbon limitation, and Degree stands for nitrogen limitation.

## 4 Discussion

### 4.1 Impact of exogenous copper on carbon metabolism

Previous studies have demonstrated that soil microbial communities experience reduced functional diversity when exposed to heavy metal contamination, such as that from mine tailings (Song et al., 2017). Additionally, the number of microbes utilizing carbon as an energy source decreases, along with their ability to efficiently use carbon substrates (Teng et al., 2008; Song et al., 2017). Our study aligns with these findings, as we observed decreased metabolic activity, reduced carbon source utilization capacity, and alterations in richness (*S*), Shannon index (*H*), and Simpson index (*D*) under Cu stress. Copper inhibits the ability of microorganisms to secrete extracellular enzymes, leading to a diminished ability to acquire essential resources. Consequently, this reduction in enzymatic activity results in decreased metabolic efficiency and carbon source utilization by microorganisms, as evidenced by ecological enzyme data. Specifically, the activities of BG, CBH, LAP, NAG, and AP were decreased with the increase of exogenous Cu content (Figure 2A). Compared with other carbon sources, the carbon source utilization of carbohydrates and amino acids was higher in each heavy metal treatment (Figure 1B). Moreover, the ability of microorganisms to use carbon sources was decreased in high Cu concentration (Cu600). This might be because the microbes need to consume more energy to maintain their normal life activities under heavy metal stress, leading to an increase in energy/carbon consumption, as reported by Xu et al. (2021) and Tao et al. (2022). Overall, heavy metal stress disrupts the diversity and carbon metabolism function of soil microbial communities (Song et al., 2017), resulting in a simplified microbial structure with a singular function (Teng et al., 2008; Wang et al., 2023). In soil with high concentrations of heavy metal pollution, certain resilient microbes become the dominant flora community (Teng et al., 2008; Qi et al., 2022).

### 4.2 Copper induces carbon and nitrogen limitation

Nitrogen limitation is widespread in terrestrial soils (Lebauer and Treseder, 2008). A lower soil C:N ratio will lead to microbes experiencing greater constraints from carbon availability than nitrogen availability, particularly when the ratio falls below 13–15 (Hodge et al., 2000; Cheng et al., 2019). Our study revealed that the C:N ratios of the selected *P. notoginseng* cultivation soils were all under 13 (as shown in Figure 2B and Table 4). Also, it was observed that the soil microbiota in *P. notoginseng* planting areas were concurrently limited by both carbon and nitrogen (as showed in Table 4). The microbial communities in the ecosystem may transit from N limitation (C saturation) to C limitation (N saturation) (Kopáček et al., 2013). Consequently, when nitrogen limitation is mitigated at low Cu concentrations (<100 mg·kg^−1^), carbon limitation is also alleviated (Allison et al., 2010) at 400 mg·kg^−1^ Cu concentration. The increasing costs of enzyme production reduce adaptation because those resources cannot be allocated to reproduction (Hall et al., 2018). Our study also found that the Pielou index is a major factor in carbon limitation, and MBN is a major factor in nitrogen limitation (Table 5) by multiple linear stepwise regression analysis. Previous studies have shown that the resource benefits of enzyme production can be invested in reproductive effort, thereby increasing microbial fitness, which is more advantageous for soil microbes in resisting Cu-induced stress (Qi et al., 2023). During the process of gene transcription and expression, soil microbes necessitated not only phosphorus but also nitrogen, which resulted in exacerbated nitrogen limitation. This result was proved by the “Evolutionary Economic Principle” by Allison in 2010 (Allison et al., 2010).

### 4.3 Exogenous copper effects on CUE of *P. notoginseng* planting soil

The Biogeochemical Equilibrium Model was used to explore the relationship between the effectiveness of environmental nutrients, EEAs, and microbial growth efficiency. On a global scale, the enzyme activity ratio for carbon, nitrogen, and phosphorus cycling is ~1:1:1 (Sinsabaugh et al., 2009). Further, Burns et al. (2013) proposed the “resource allocation theory,” which highlights the coupling relationship between resource effectiveness in soil and the relative activity of soil enzymes. The microbes will allocate more energy (C) and nutrients (N, P) to produce limited enzymatic resources as the environmental substrate is abundant and effective resources are scarce. In our study, the enzyme activity ratios related to soil carbon, nitrogen, and phosphorus cycling were ~1:1.5:1 (control), 1:1.2:1 (Cu100), 1:1.4:1 (Cu400), and 1:2.4:1 (Cu600). The average values of 40 major terrestrial soils in the globe were 1.41 for the enzyme C:N value, 0.62 for the C:P value, and 0.44 for the N:P value (Burns et al., 2013). Besides, the enzyme C:N values for the four treatments in our experiment were found below the average values of the 40 major terrestrial soils. The enzyme C:N values for all the treatments were estimated at 0.699 in the control, 0.808 in the Cu100, 0.748 in the Cu400, and 0.402 in the Cu600. It indicated that the microbial community in *P. notoginseng* plantation soil was limited by carbon and nitrogen.

The CUE may be relatively high when carbon availability (i.e., energy) is limited (Blagodatskaya and Kuzyakov, 2008). Furthermore, microbes may guide carbon into overflow respiration, leading to a decrease in CUE when nutrient limitation occurs (Manzoni et al., 2012; Sinsabaugh et al., 2013). Our study identified key factors influencing CUE, including MBC, AP, C:P ratio, and LAP. The production of extracellular enzymes by microbes requires carbon, which may lead to additional respiratory costs (Cotrufo et al., 2013; Moorhead et al., 2015), ultimately reducing CUE (Malik et al., 2018). In addition, temperature and soil moisture can alter microbial metabolism, affecting the balance between growth (μ) and assimilation rates, thus impacting CUE (Sinsabaugh et al., 2016). The decrease in CUE may result from the coincident limitation of the two elements (Waring et al., 2014; Malik et al., 2018). In our study, the *P. notoginseng* plantation soil selected was severely limited by carbon and nitrogen, leading to a decrease in CUE. Microbes may lower their CUE to buffer against environmental pressures because maintaining the microbe's condition requires more energy (Soares and Rousk, 2019). However, the soil microbial subjected to Cu stress increased CUE due to decreased microbial quantity. Environmental factors affect the ability of soil microbial communities to obtain energy and nutrients, as well as the availability of soil nutrient forms. These results are confirmed by Qi et al.'s (2016) study, which states that unstable carbon in the soil varies continuously with temperature, affecting the activity of EEAs by controlling soil microbial decomposition rates and mineralization processes. Our study found that CUE was mainly influenced by MBC, AP, C:P, and LAP from multiple linear stepwise regression analyses.

The enzymes that obtain nutrients (N and P) are equally important for the effect of CUE. Heavy metals affect the metabolic function of soil microbes by changing the rate of decomposition and diminishing the ability of microbes to secrete extracellular enzymes (Gong et al., 2019). Therefore, their ability to acquire energy (C) and nutrients (N) from the external environment is reduced (Duan et al., 2022). According to Allison's theory (Allison et al., 2010), the increased production costs for enzymes reduced adaptability because these resources cannot be allocated to reproduction. The positive impact of microbial biomass on carbon and nitrogen limitation might be because soil microbes do not produce more extracellular enzymes, which can obtain energy (C) and nutrients (N and P) when subjected to Cu stress (Manzoni et al., 2012; Burns et al., 2013; Tao et al., 2022). This is detrimental to producing microbial Cu resistance genes to resist external stress. The increase in microbial carbon/nitrogen ratio influenced the increase of CUE (Sinsabaugh et al., 2013). The allocation of resources for nitrogen acquisition by microbes reduced CUE (Cleveland and Liptzin, 2007), which led to a positive effect from MBC, MBN, and MBP, while microbial diversity had a negative effect on CUE.

## 5 Conclusions

The current study revealed that soil microbes under high Cu concentration reduced metabolic activity and the diversity indices of the microbial community; however, the low Cu concentration has a stimulating effect on microbial mass and diversity (such as the Shannon and Simpson indices).

The enzyme stoichiometry analysis revealed that microbes in PNG planting soils were both subjected to carbon and nitrogen limitations. In Cu-treated soils, limitations of carbon and nitrogen were ameliorated at low concentrations (<100 mg·kg^−1^). Whereas high concentrations (up to 600 mg·kg^−1^) presented more pronounced carbon and nitrogen restrictions than the control. Under elevated Cu stress conditions, the capacity of soil microbes to obtain energy (C) and nutrients (N) was substantially impeded, consequently reducing microbial diversity. Environmental factors, including pH, MBP, and TP, significantly influence the metabolism and EEAs of soil microbial communities. Heavy metal stress can also reduce EEAs, as the increased production cost of enzymes reduces adaptability to the growth environment. PLS-SEM pathway analysis showed that soil carbon and nitrogen limitation is aggravated by soil microbial mass and microbial diversity (0.654 and 0.424), and soil element and stoichiometric ratios and EEAs relieve soil carbon and nitrogen limitation (0.527 and 0.589). Soil element stoichiometry and microbial mass had a positive effect on CUE (−1.490).

## Data availability statement

The data supporting the conclusions of this article will be made available by the authors, without undue reservation. Requests to access the datasets should be directed to: fangling.fan@ynnu.edu.cn.

## Author contributions

TW: Software, Writing – original draft, Writing – review & editing. XW: Methodology, Writing – review & editing. TH: Writing – review & editing. XM: Resources, Writing – review & editing. HY: Writing – review & editing. ZT: Resources, Supervision, Writing – review & editing. FF: Resources, Supervision, Writing – review & editing. YH: Resources, Supervision, Writing – review & editing.
