# Supplementary figures and images for: Effects of exogenous copper on microbial metabolic function and carbon use efficiency of Panax notoginseng planting soil
Source: Front Microbiol. 2024 Jul 10;15:1390921. doi: 10.3389/fmicb.2024.1390921 (PMC11266184; doi:10.3389/fmicb.2024.1390921)

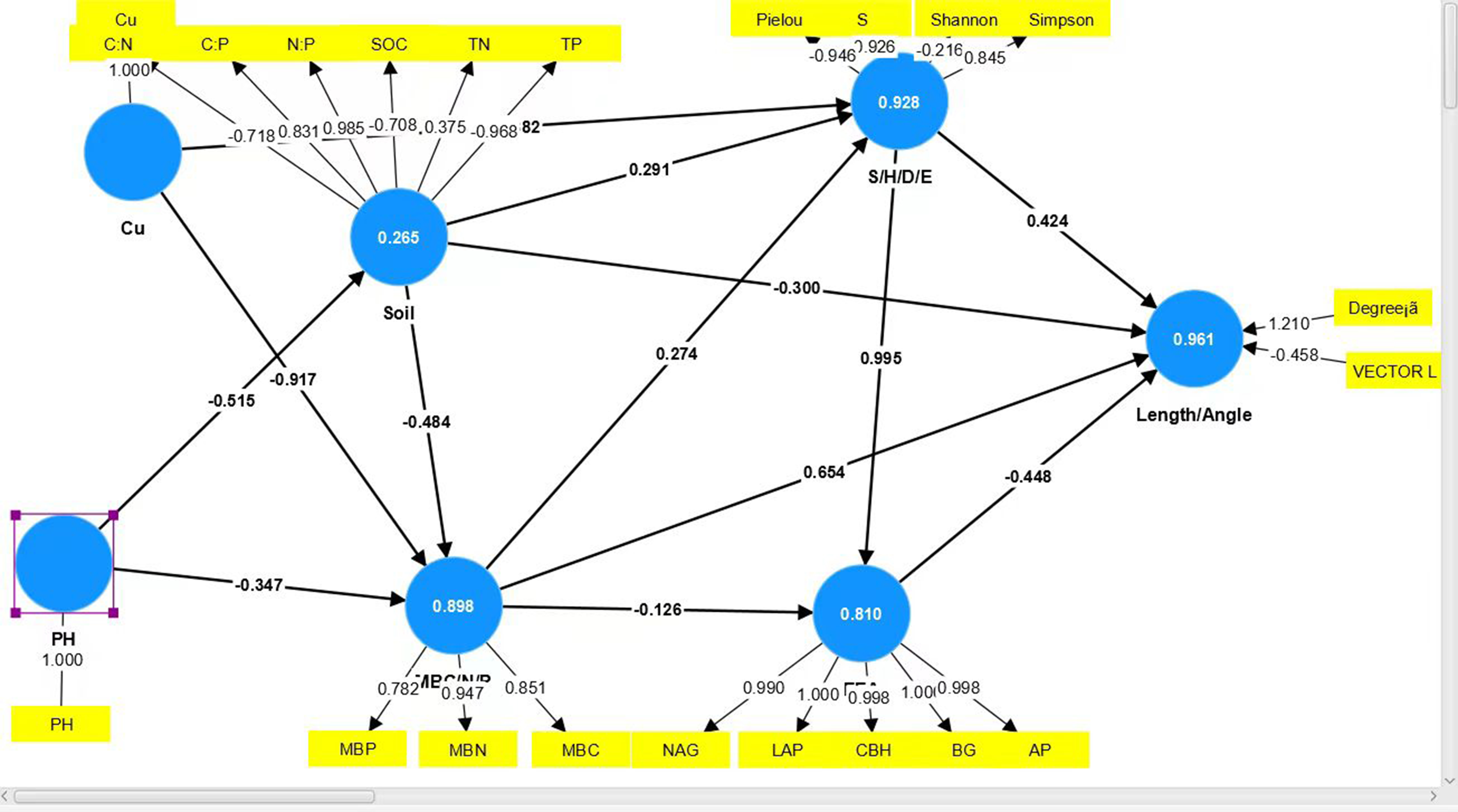

Supplement: Supplementary file 2 [file Image_1.JPEG]

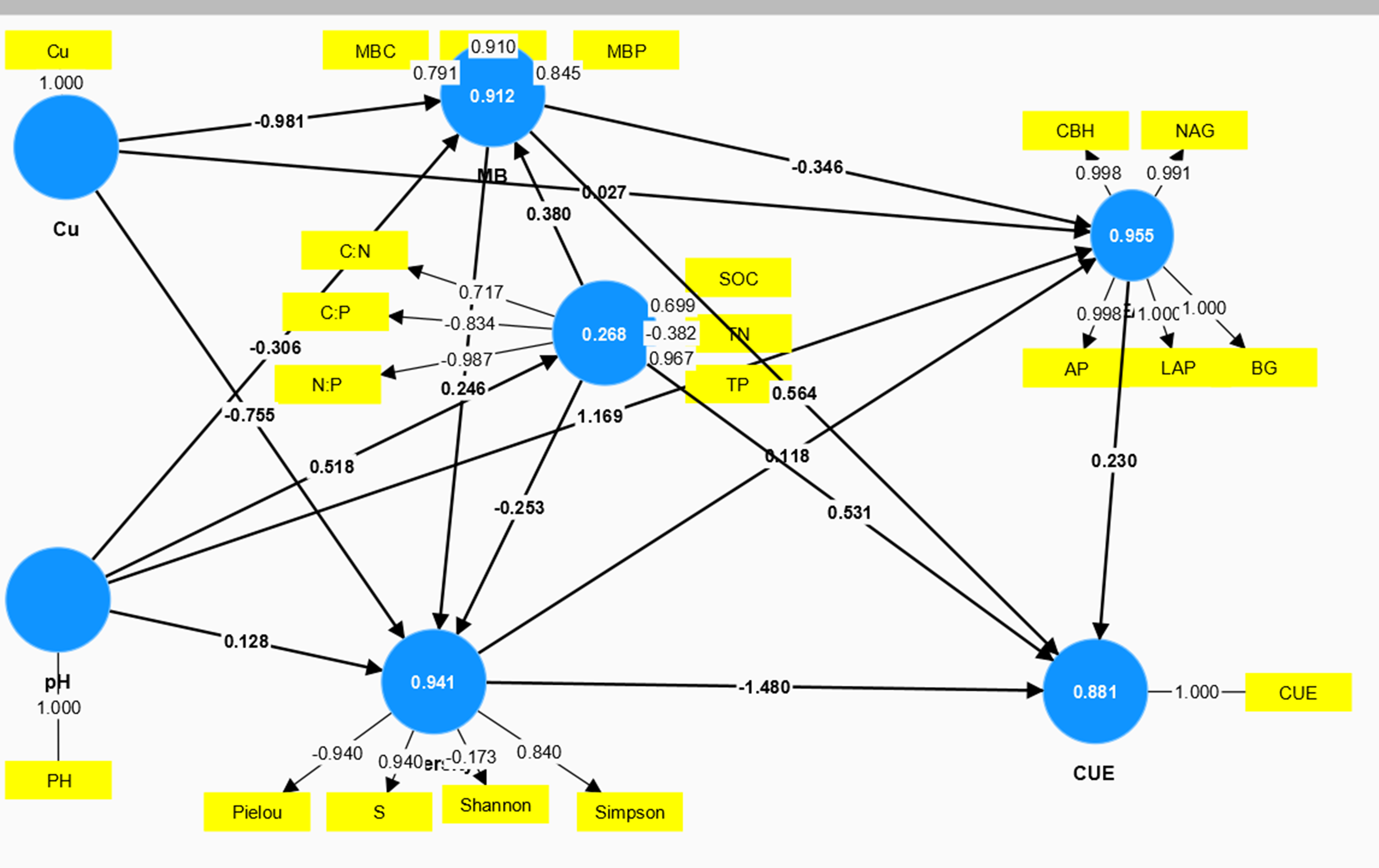

Supplement: Supplementary file 3 [file Image_2.PNG]
